# Age of diagnosis for children with chromosome 15q syndromes

**DOI:** 10.1186/s11689-023-09504-x

**Published:** 2023-11-07

**Authors:** Anne C. Wheeler, Marie G. Gantz, Heidi Cope, Theresa V. Strong, Jessica E. Bohonowych, Amanda Moore, Vanessa Vogel-Farley

**Affiliations:** 1 Genomics, Ethics, and Translational Research Program, RTI International, 3040 W. Cornwallis Rd, Research Triangle Park, NC 27709 USA; 2 Foundation for Prader Willi Research, Covina, CA USA; 3 Angelman Syndrome Foundation, Aurora, IL USA; 4 Dup15q Alliance, Matthews, NC USA

## Abstract

**Objective:**

The objective of this study was to identify the age of diagnosis for children with one of three neurogenetic conditions resulting from changes in chromosome 15 (Angelman syndrome [AS], Prader-Willi syndrome [PWS], and duplication 15q syndrome [Dup15q]).

**Methods:**

Data about the diagnostic process for each condition were contributed by the advocacy organizations. Median and interquartile ranges were calculated for each condition by molecular subtype and year. Comparison tests were run to explore group differences.

**Results:**

The median age of diagnosis was 1.8 years for both AS and Dup15q. PWS was diagnosed significantly younger at a median age of 1 month. Deletion subtypes for both PWS and AS were diagnosed earlier than nondeletion subtypes, and children with isodicentric duplications in Dup15q were diagnosed earlier than those with interstitial duplications.

**Conclusion:**

Understanding variability in the age of diagnosis for chromosome 15 disorders is an important step in reducing the diagnostic odyssey and improving access to interventions for these populations. Results from this study provide a baseline by which to evaluate efforts to reduce the age of diagnosis for individuals with these conditions.

**Supplementary Information:**

The online version contains supplementary material available at 10.1186/s11689-023-09504-x.

## Article summary

Chromosome 15 imprinting disorders have different phenotypes and diagnostic pathways despite similar genetic origins. In this study the age of diagnosis for Angelman, Prader-Willi, and Dup15q syndromes was compared across molecular subtypes over the past 10 years. Results suggest that infants with PWS are typically diagnosed within the first 6 months of life, whereas children with As or Dup15q do not receive a diagnosis until closer to 24 months.

## Introduction

Earlier identification of neurogenetic conditions is an important endeavor to improve the quality of life and long-term outcomes for individuals with the conditions, their families, and caregivers. Delayed diagnoses reduce access to early behavioral and medical interventions, prohibit surveillance for potential disease manifestations, potentially increase the severity of comorbid conditions (e.g., autism, epilepsy), and can cause an emotionally and financially challenging diagnostic odyssey for the family [1]. Early identification becomes even more critical with the potential of gene therapies that may improve outcomes or even “cure” neurogenetic conditions. These treatments hold promise to have a profound impact on the quality of life for those currently living with a neurogenetic condition at any stage of life. However, to achieve the maximum benefit, it is likely that treatment should be initiated prior to the onset of symptoms, which, depending on the condition, is likely to occur very early in life, or even during the prenatal period [2].

Prader-Willi (PWS), Angelman (AS), and duplication 15q (Dup15q) syndromes are conditions with distinct phenotypes but similar molecular origins—all originating from abnormalities within the same region at chromosome 15q11.2-q13.1. Although all three conditions have an inherited molecular subtype, the majority of cases result from de novo gene changes. Genes implicated in these three conditions are all targets for gene therapy trials, and as a result, patient advocacy organizations, clinicians, and basic scientists have combined efforts to improve therapeutic development and diagnostic processes.

*Prader-Willi Syndrome* occurs as a result of loss of expression of genes on the *paternally* inherited chromosome 15q11.2-q13. The most common mechanism resulting in PWS is a deletion of the 15q11-q13 imprinted region (60–65% of patients); maternal uniparental disomy (UPD) occurs in approximately 35% of patients; the remaining ~ 3% have an imprinting defect (ID).

The typical PWS phenotype includes hypotonia, short stature, small hands and feet, and mild to moderate intellectual disability. Behavioral issues, including symptoms of anxiety, temper outbursts, and deficits of social cognition, are also present in many individuals with PWS. Significant hypotonia at birth and feeding difficulties can lead to failure to thrive in infancy. This difficulty with feeding generally gives way to hyperphagia in early childhood, which, if not controlled, will lead to obesity and associated health issues.

Hypotonia and feeding difficulties in infancy, along with dysmorphology that is sometimes present at birth, are often causes for referral for genetic testing, thereby making PWS the most likely of the three C15 conditions to be diagnosed in the newborn period. However, infants with milder hypotonia or less common subtypes (UPD, ID) may be more likely to be diagnosed later in childhood [3].

Current first-line treatment for PWS includes growth hormone, which is most effective if initiated between 4 and 24 months of age, with physical and speech therapy starting as soon as possible. Novel therapies currently in development for PWS include those that may prove most effective when administered in the newborn period, such as oxytocin [4]. As more studies indicate more impactful outcomes for those started on treatment in early infancy, there will be increasing emphasis on earlier diagnosis. Early identification to optimize parent education and allow close monitoring of diet and behavior management strategies are also recommended.

*Angelman syndrome* occurs with the loss of expression from the *maternally* inherited *UBE3A* gene. In nearly three-quarters of patients, this is the result of deletion of maternal chromosome 15q11.2-q13 region. A pathogenic variant in the maternally derived *UBE3A* gene occurs in around 11% of patients, while paternal UPD occurs in around 8% and an imprinting defect in around 7% [5].

The phenotype of AS is characterized by severe to profound intellectual disability, minimal or absent verbal speech, seizures, ataxia, and an easily excitable, happy demeanor. Symptoms of anxiety, short attention span, and difficulty with sleep are also commonly experienced. Although symptoms of AS often begin within the first year of life, symptoms are not obvious at birth and early features can be mistaken for other forms of developmental delay (e.g., autism, cerebral palsy [6]).

The treatment landscape for AS has changed dramatically over the last decade. Historically, the only treatment options were symptom-based (e.g., seizure medication, behavior management), and new discoveries in *UBE3A* function and mechanisms for gene therapy now suggest that disease-modifying therapies may be imminent. Clinical trials testing the safety and efficacy of these therapeutics are currently in progress; positive results are likely to rapidly lead to increased urgency in earlier identification to maximize treatment outcomes.

*Dup15q syndrome* also involves the PWS/AS critical region, but in contrast to PWS and AS, it is caused by overexpression of genes within the region, usually the result of at least one extra maternally derived copy of the region. In isodicentric Dup15q (Idic15; the most common of two forms), a small supernumerary chromosome with two extra copies of the maternal 15q11.2-q13 region is present in addition to two normal chromosomes 15. This results in individuals having three maternal copies and one paternal copy of the locus. The other form of Dup15q, maternal interstitial duplication, involves one extra copy of the maternal 15q11.2-q13 region, resulting in two maternal copies and one paternal copy of the locus. Paternally derived duplications of 15q11.2-q13 have also been reported, although the resulting phenotype is variable and less well characterized [7].

Of the three C15 conditions, Dup15q is thought to have the most heterogeneous presentation, which may contribute to later diagnoses [8]. However, primary features overlap with both AS and PWS, including hypotonia, mild to severe intellectual disability, seizures, and high rates of comorbid autism. Several studies have indicated that individuals with Idic15 have more severe phenotypes than those with interstitial duplications [8, 9].

In part because of the molecular overlap, diagnostic and treatment efforts for Dup15q overlap significantly with efforts for AS and PWS. However, primary treatment recommendations currently are primarily symptom-focused (e.g., seizure management, behavioral/educational therapies). Drug discovery and gene therapy efforts are underway for Dup15q but are further behind than those for AS or PWS.

### Diagnostic processes

Although guidelines exist from professional organizations such as the American Academy of Pediatrics and the American College of Medical Genetics, there remain inconsistencies in how and when children are referred for genetic testing. Diagnostic evaluations of young children with a C15 condition nearly always result from the onset of symptoms, but the process can vary significantly based on the condition, clinical presentation, molecular subtype, and knowledge on the part of the evaluating healthcare professional. For some individuals, PWS or AS may be in the differential because of a phenotypic presentation consistent with the diagnosis. The stepwise diagnostic approach for these patients can differ from clinic to clinic, but typically begins with DNA methylation testing via PCR, Southern hybridization, or MS-MLPA chromosome 15 testing [10]. An abnormal methylation result is diagnostic of AS or PWS but will require additional testing to characterize the underlying molecular subtype. Not all individuals with suspected AS will have abnormal DNA methylation results, and in these individuals, *UBE3A* sequencing is necessary. In PWS, nearly all (> 99%) will be positive based on DNA methylation analysis.

For individuals with a C15 disorder who present with nonspecific features that are not clearly suggestive of PWS or AS, such as developmental delay, seizures, mild hypotonia, or autism, chromosome analysis or chromosomal microarray (CMA) may be ordered as first-tier diagnostic tests [11, 12]. CMA utilizing either oligonucleotide or single-nucleotide polymorphism (SNP) probes will detect Dup15q but cannot differentiate between isodicentric chromosome 15 and an interstitial duplication. Chromosome analysis is needed to identify the supernumerary isodicentric chromosome 15 but is not able to identify most interstitial duplications seen in individuals with Dup15q. Therefore, to achieve an accurate molecular diagnosis, a combination approach including both chromosome analysis and CMA is needed. Because the phenotype may differ depending on the parent of origin, methylation analysis can be pursued to determine if the duplication was maternally or paternally derived [13].

CMA will also detect chromosome 15 deletions associated with AS and PWS. However, only SNP arrays have the ability to detect loss of heterozygosity, which can identify patients with chromosome 15 uniparental disomy (UPD15) resulting from segmental or total isodisomy [13]. Thus, SNP arrays will not be diagnostic for patients with UPD15 because of heterodisomy, nor for those with imprinting centers due to epimutations or microdeletions below the level of resolution for CMA. Patients who remain undiagnosed after CMA may go on to receive additional testing until a diagnosis is eventually established.

This diagnostic odyssey, which mirrors the experiences of many individuals with rare neurogenetic conditions, may take years, which can result in high financial and emotional stress for families and delayed start of treatment for the children. Identifying current trends in the age of diagnosis for a condition can help to establish strategies for reducing delays and potentially improving prognoses. The goal of the current study was to describe the distribution of age of diagnosis for C15 conditions, including differences within and across conditions and potential trends across the last 10 years.

## Methods

This study was reviewed by RTI International’s Institutional Review Board and deemed exempt. Data that included questions about the diagnostic process were obtained from participants registered with the *Angelman Syndrome Foundation*, the Global Prader-Willi Syndrome Registry (sponsored by the *Foundation for Prader-Willi Research*), or the *Dup15q Alliance*. Questions were asked as part of registry enrollment; participants were directed to the patient advocacy organizations by medical professionals or through internet searches. Data were deidentified prior to transfer. Participants diagnosed between 2010 and 2021 for PWS and AS, and between 2010 and 2018 for Dup15q, were included in the analysis. For PWS and Dup15q, the year of diagnosis was estimated based on birth year and age of diagnosis.

### Statistical analysis

For each syndrome, the genetic subtype and sex of registry participants were summarized as counts and percentages. Age of diagnosis was described as median and interquartile range (IQR), and distributions by year were examined using box plots, with age truncated at 15 years for display purposes. For PWS and AS, the age of diagnosis was calculated in fractions of a year for ages up to 2 years and in whole years thereafter. For Dup15q, age of diagnosis up to 2 years of age was reported in 3-month intervals (i.e., 1–3 months, 4–6 months), and after age 19 was reported in 10-year intervals (i.e., 20–29 years) so the mid-point of the interval was used for analysis. Prenatal diagnoses were considered to have occurred at 0 months for analysis purposes. Age of diagnosis was compared between syndromes and between genetic subtypes and sexes within each syndrome using pairwise Wilcoxon rank sum tests, and differences between the distributions were estimated as Hodges-Lehmann location shift and 95% confidence intervals. For each syndrome, the Spearman correlation was used to assess whether the age of diagnosis increased or decreased over time.

## Results

Data were included for 350 individuals with PWS, 1241 with AS, and 217 with Dup15q, and participant characteristics are shown in Table 1 and Supplemental Table S1.

**Table 1:** Characteristics of participants diagnosed between 2010 and 2021 for PWS and AS, and between 2010 and 2018 for Dup15q

Variable	Category	PWS (*N* = 350)	AS (*N* = 1241)	Dup15q (*N* = 217)
Genetic subtype	Deletion	181 (51.7%)	629 (50.7%)	n/a
	*UBE3A* variant	n/a	213 (17.2%)	n/a
	Uniparental disomy	117 (33.4%)	96 (7.7%)	n/a
	Imprinting defect	9 (2.6%)	25 (2%)	n/a
	Other	7 (2%)	10 (0.8%)	*
	Isodicentric	n/a	n/a	102 (47%)
	Interstitial	n/a	n/a	82 (37.8%)
	Other or Unknown	36 (10.3%)	268 (21.6%)	33 (15.2%)*
Male		177 (50.6%)	666 (53.7%)	122 (56.2%)
Age of diagnosis (years)	Median [IQR]	0.1 [0, 0.2]	1.8 [1.1, 3]	1.8 [0.9, 4]

^*^The Dup15q registry lumped other or unknown together

**Fig. 1:**
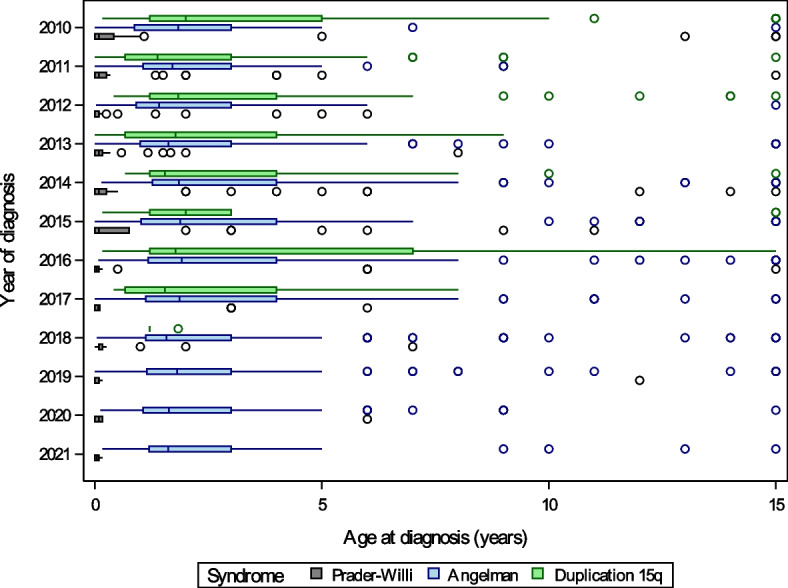
Age at diagnosis for Prader-Willi syndrome, Angelman syndrome, and duplication 15q registry participants diagnosed between 2010 and 2021 for PWS and AS, and between 2010 and 2018 for Dup15q. Boxes represent the interquartile range, with the median shown as a vertical line. Whiskers extend to values close enough not to be considered outliers (within 1.5 times the interquartile range) and circles denote outliers. For display purposes, ages greater than 15 years were set equal to 15 years

**Fig. 2:**
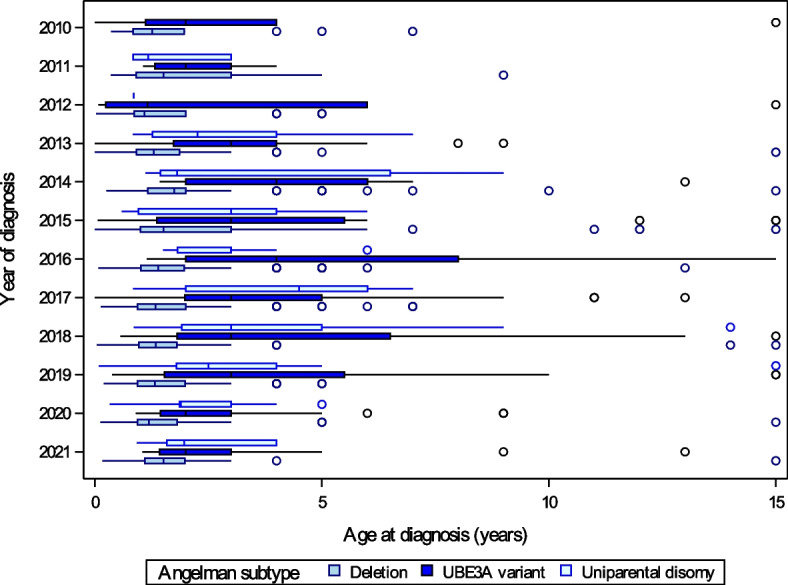
Age at diagnosis for deletion, UBE3A variant, and uniparental disomy genetic subtypes of Angelman syndrome registry participants diagnosed between 2010 and 2021. Boxes represent the interquartile range, with the median shown as a vertical line. Whiskers extend to values close enough not to be considered outliers (within 1.5 times the interquartile range) and circles denote outliers. For display purposes, ages greater than 15 years were set equal to 15 years

**Fig. 3:**
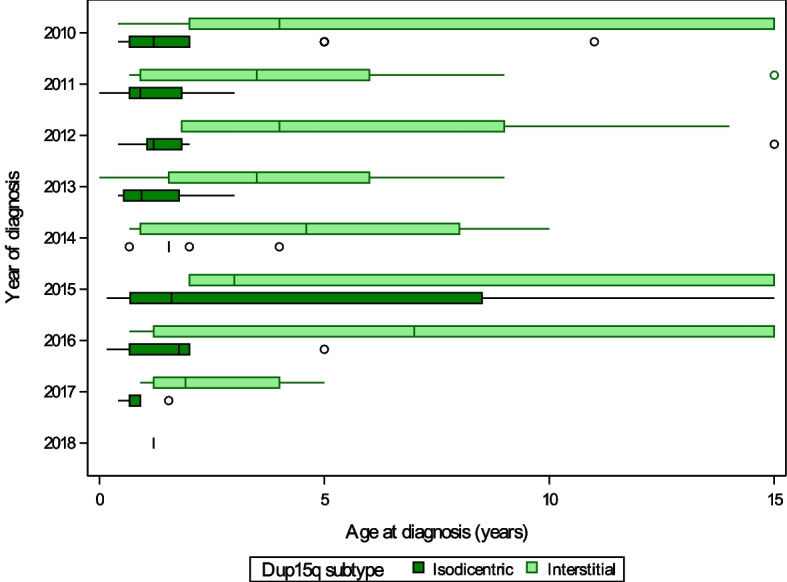
Age at diagnosis for isodicentric and interstitial duplication subtypes of duplication 15q registry participants diagnosed between 2010 and 2018. Boxes represent the interquartile range, with the median shown as a vertical line. Whiskers extend to values close enough not to be considered outliers (within 1.5 times the interquartile range) and circles denote outliers. For display purposes, ages greater than 15 years were set equal to 15 years

Age of diagnosis across conditions and molecular subtypes between are illustrated by year of diagnosis (2010–2021) in Figs. 1, 2, and 3 and year of birth (1990–2021) in Supplemental Figures S1, S2 and S3. PWS was diagnosed significantly earlier than AS or Dup15q, with 66% of PWS diagnoses occurring within a month of birth and 85% by 1 year compared to a median [IQR] age of diagnosis of 1.8 years [0.9, 4] for Dup15q (location shift − 1.5 [95% CI − 1.7 to − 1.2], *p* < 0.001) and 1.8 years [1.1, 3] for AS (location shift − 1.4 [95% CI − 1.6 to − 1.3], *p* < 0.001) (Fig. 1). For PWS, there was a small but statistically significant difference in age of diagnosis between the deletion and UPD subtypes (deletion 0 years [0, 0.1], UPD 0.1 years [0, 0.2], location shift 0 [95% CI − 0.1 to 0], *p* = 0.003). For AS, the diagnosis was earlier in patients with deletion (1.4 years [1, 2]) compared to *UBE3A* variants (3 years [1.6, 5], location shift − 1.2 [95% CI − 1.5 to − 1], *p* < 0.001), and UPD (2.5 years [1.4, 4], and location shift − 1 [95% CI − 1.2 to − 0.6], *p* < 0.001) (Fig. 2). Dup15q diagnosis was earlier in those with isodicentric (1.2 years [0.7, 2]) versus interstitial duplications (4 years [1.8, 8], location shift − 2 [95% CI − 3 to − 1.1], *p* < 0.001) (Fig. 3). Prenatal diagnosis was reported for 3 PWS, 11 Angelman, and 8 Dup15q participants. Age of diagnosis did not differ by sex for any of the syndromes, and there was no correlation between age and year of diagnosis over the period 2010–2021 (− 0.1 < *r* < 0 for all syndromes). Examination of age of diagnosis by year of birth supported the finding that PWS diagnosis generally occurred within the first year of life for individuals born in 2010 or later, while age of diagnosis was more variable for those born with AS or Dup15q during the same period (Supplemental Figures S1, S2 and S3).

## Discussion

Results from this secondary data analysis suggest that C15 conditions are usually diagnosed within the first 3 years of life, but timing differs significantly based on condition and molecular subtype. The youngest and most consistently diagnosed in infancy of the three conditions was PWS, with 66% diagnosed by 1 month. This is likely because of symptoms of hypotonia and feeding difficulties that are present at birth in newborns with PWS, serving as “red flags” that can lead to extended hospital stays with diagnostic tests run in the first few weeks of life. Although the age of diagnosis was higher in individuals with PWS due to UPD compared to deletion, more than three-quarters of individuals in both groups were diagnosed in 3 months, which is likely because of the methylation testing identifying all subtypes of PWS.

In contrast, children with AS were diagnosed at a median age of around 2 years, significantly older than those with PWS. Children with AS are likely not assessed until they miss motor or communication milestones, usually toward the end of the first year of life. Notably, children with a *UBE3A* variant or UPD were diagnosed significantly later than those with a deletion. This may be because these subtypes require additional testing, which would inherently result in later diagnoses. In addition, most studies comparing phenotypes of children with deletion and nondeletion subtypes of AS have indicated higher cognitive functioning in those with nondeletion subtypes [5, 14, 15] which also may contribute to later diagnoses.

Children with Dup15q were diagnosed at a similar age to those with AS and significantly older than those with PWS. Similar to children with AS, the diagnostic process for children with Dup15q likely begins when they miss motor milestones or when they have their first seizure. Infantile spasms are relatively common in Dup15q, a symptom which may result in an earlier diagnosis but also have significant negative long-term impacts for the child [16]. Not surprisingly, children with the Idic15 form of Dup15q, who are typically more severely impacted, were diagnosed significantly younger than those with interstitial duplications.

The last decade has seen a substantial increase in research and knowledge about chromosome 15 conditions. In addition, there has been a greater emphasis on earlier identification of neurogenetic conditions, including American Academy of Pediatrics guidelines published in 2013 that recommend genetic testing for any child who presents with intellectual or developmental delays [17]. Despite these initiatives, we found that the age of diagnosis for C15 syndromes has not decreased in the last decade. Continued emphasis on educating medical professionals about the phenotypes of these conditions is important, as are the refinement and expansion of tools like FindZebra or Orphenet, which catalog and help physicians identify rare conditions based on symptom presentation. But these initiatives, at best, can reduce the amount of time between first concerns and diagnoses; they cannot help identify infants before symptoms occur. Given that emerging therapeutics are believed to be most effective if initiated pre-symptomatically [2], it is important to continue seeking ways to reduce the age of diagnosis even more.

The promise of gene therapies requires a focused effort to identify and diagnose conditions as early as possible. However, beyond invasive prenatal treatment, which has several ethical and logistical challenges, the best hope for maximizing outcomes would be treatment shortly after birth. Neonatal diagnoses of conditions are primarily made through state-mandated newborn screening (NBS) panels. The decision regarding which conditions are included on NBS panels is made first at the national level, by the Advisory Committee on Heritable Disorders in Newborns and Children, which considers how well-nominated conditions meet rigorous criteria for the Recommended Uniform Screening Panel. These criteria encompass the overall net benefit of screening, which includes the health of the child and certainty of evidence regarding the benefit of early identification and the capability of state NBS programs to conduct the screening [18].

C15 conditions do not currently meet eligibility criteria for these NBS programs, primarily because there are no reliable screening tests or limited proven treatments for the conditions. The landscape of NBS for C15 conditions is rapidly shifting, however. For the purposes of NBS, methylation analysis has the highest diagnostic yield and is feasible to perform on dried blood spots [19, 20]. Methylation-based processes and assays have been developed and appear to have good reliability and sensitivity, although more testing is needed [21]. This, along with emerging therapeutic development may see these, and other rare conditions, prioritized for earlier identification, including expanded NBS panels.

A limitation of this study is that we did not have information about the emergence of symptoms or the age of entry into early intervention. Nor do we know the experiences of caregivers in getting to the point of diagnosis. This would be helpful for determining the extent to which delayed diagnoses impact child or family outcomes. This study also relied primarily on parent reports of the diagnostic timing; formal documentation of the timing of the diagnosis was not provided. We examined the age of diagnosis by year of diagnosis as well as year of birth, and there are limitations to both approaches. Specifically, when comparing the age of diagnosis across years of diagnosis, the distribution of age at diagnosis will be skewed upward by individuals who were diagnosed later in life because they were born in years when early diagnosis was less common. In contrast, the distribution of age of diagnosis will be skewed downward when compared across years of birth, since the only diagnoses reported for those born in later years will necessarily have happened at earlier ages. We examined the data both ways, and the findings were consistent across the two approaches.

Further, by definition, the population that we report on here are all individuals who had received a diagnosis. We do not know how many individuals remain undiagnosed or misdiagnosed, nor do we have a full appreciation for how the diagnostic process may differ for individuals from different race, ethnic, or socioeconomic backgrounds. For example, PWS deletions are often associated with decreased *OCA2* gene expression, leading to a “blonde hair, blue-eyed” phenotype, even in families with typically darker features. This may result in underdiagnoses among African and Hispanic Americans who do not “look” like the children with PWS presented in most textbooks. Of the three chromosome 15 syndromes, we are only able to report the race and ethnicity of PWS registry participants (Supplemental Table S1). In this group, race was race largely White (56%) or unknown (29%) and ethnicity was mostly non-Hispanic/Latino (53%) or unknown (39%) which may reduce the generalizability of the data to the broader PWS population.

## Conclusions

Despite these limitations, this study provides an important contribution to our understanding of the diagnostic process for children with a chromosome 15 condition. This information can be used to help inform efforts to educate pediatricians and other front-line providers on the features of these conditions to promote earlier screening. Further, this paper provides baseline data on the diagnostic processes that can be used to measure the efficacy of efforts designed to improve earlier diagnoses.

To our knowledge, this is the first study to document the age of average diagnosis for these conditions, an important first step in reducing the duration of diagnostic odysseys and improving long-term outcomes for individuals with one of these conditions and their families.

### Supplementary Information

**Additional file 1: Table S1.** Race and Ethnicity of Registry Participants Diagnosed with Prader Willi Syndrome in 2010-2021. **Figure S1.** Age at Prader Willi Syndrome Diagnosis for Registry Participants Born in 1990-2021. **Figure S2.** Age at Angelman Syndrome Diagnosis for Registry Participants Born in 1990-2021. **Figure S3.** Age at Duplication 15q Syndrome Diagnosis for Registry Participants Born in 1990-2017.

## Data Availability

Data are available through the advocacy organizations, by request and with IRB approval.

## Abbreviations

AS: Angelman syndrome
C15: Chromosome 15
CMA: Chromosomal microarray
Dup15q: Duplication 15q syndrome
EI: Early intervention
Idic15: Isodicentric Dup15q
ImpD: Imprinting defect
IQR: Interquartile range
MS-MLPA: Methylation-specific multiplex ligation-dependent probe amplification
NBS: Newborn screening
PCR: Polymerase chain reaction
PWS: Prader-Willi syndrome
SNP: Single-nucleotide polymorphism
UPD: Uniparental disomy
