# Supplementary material for: Age of diagnosis for children with chromosome 15q syndromes
Source: J Neurodev Disord. 2023 Nov 7;15:37. doi: 10.1186/s11689-023-09504-x (PMC10629121; doi:10.1186/s11689-023-09504-x)
Supplement: Supplementary file 1 — Additional file 1: Table S1. Race and Ethnicity of Registry Participants Diagnosed with Prader Willi Syndrome in 2010-2021. Figure S1. Age at Prader Willi Syndrome Diagnosis for Registry Participants Born in 1990-2021. Figure S2. Age at Angelman Syndrome Diagnosis for Registry Participants Born in 1990-2021. Figure S3. Age at Duplication 15q Syndrome Diagnosis for Registry Participants Born in 1990-2017. [file 11689_2023_9504_MOESM1_ESM.docx]

***Age of Diagnosis for Children with Chromosome 15q Syndromes***

**Supplemental Materials**

Table S1. Race and Ethnicity of Registry Participants Diagnosed with Prader Willi Syndrome in 2010-2021

| **Category** | **N (%)** |
| --- | --- |
| **Race** | |
| White | 197 (56.3%) |
| Black or African American | 8 (2.3%) |
| Asian | 12 (3.4%) |
| Multi-ethnic | 24 (6.9%) |
| Other | 7 (2%) |
| Unknown | 102 (29.1%) |
| **Ethnicity** | |
| Ashkenazi Jewish | 4 (1.1%) |
| Hispanic or Latino | 23 (6.6%) |
| Non-Hispanic or Latino | 187 (53.4%) |
| Unknown | 136 (38.9%) |

Figure S1. Age at Prader Willi Syndrome Diagnosis for Registry Participants Born in 1990-2021

Note: Age at diagnosis is truncated at 15 years for display purposes

Figure S2. Age at Angelman Syndrome Diagnosis for Registry Participants Born in 1990-2021

Note: Age at diagnosis is truncated at 15 years for display purposes

Figure S3. Age at Duplication 15q Syndrome Diagnosis for Registry Participants Born in 1990-2017

Note: Age at diagnosis is truncated at 15 years for display purposes
